# Developing CSCO Lung Cancer Practice Guidelines Stratified by Resource Availability and Treatment Value

**DOI:** 10.1200/JGO.2016.006734

**Published:** 2016-10-12

**Authors:** Qing Zhou, Yi-Long Wu

**Affiliations:** **All authors:** Guangdong Lung Cancer Institute, Guangdong General Hospital, and Guangdong Academy of Medical Sciences, Guangzhou, People’s Republic of China

China is the third-largest country by area and the largest low- to middle-income country in the world. Economic growth and urbanization have resulted in sedentary lifestyles, increasingly Western dietary habits, and increases in smoking rates, alcohol consumption, and environmental pollution. These changes have led to increased rates of noncommunicable diseases such as cancer.^1,2^ According to data from GLOBOCAN 2012 (Estimated Cancer Incidence, Mortality and Prevalence Worldwide in 2012), lung cancer accounts for 21.3% of all cancers and 27.1% of all cancer-related deaths in China, making it the most common cancer in terms of both incidence and mortality for women and men.^3^ Many lung cancer treatment guidelines have been developed, including the ASCO guidelines and National Comprehensive Cancer Network Guidelines in the United States and the European Society for Medical Oncology guidelines. Asian countries such as Japan and South Korea have their own lung cancer treatment guidelines. In contrast, diagnosis and treatment of Chinese patients with lung cancer is still mostly dependent on foreign guidelines. Given China’s vast population, wide geographic span, and diverse cultures and socioeconomic groups, cancer treatments vary greatly, and one standard set of guidelines will not suffice. Although medical care in western China has improved rapidly, the more developed eastern areas continue to benefit most from recent progress in cancer treatment.^4^ Urban residents generally have higher socioeconomic status and better access to cancer care compared with rural residents. Patients with cancer in more developed regions are more likely to have access to essential drugs, therapies, and screenings. But some patients are overtreated through the off-label use of anticancer drugs,^5^ which can occur as a result of poor socioeconomic status, shortage of anticancer drugs, profitable prescriptions, unfamiliarity with the latest medical developments, and a lack of guidelines that suit all Chinese patients with cancer. At the same time, China is one of the most active regions for lung cancer research, which is beneficial to patients with lung cancer worldwide. Expanding the benefits of lung cancer research to the entire Chinese population is one of the most prominent issues facing Chinese care providers.

Applying Western cancer research to Chinese patients is problematic. Chinese patients with non–small-cell lung cancer (NSCLC) differ from Western patients in multiple ways, including different driver mutations, different etiologies,^6-10^ and different tolerances to treatment. Therefore, several top cancer experts from the Chinese Society of Clinical Oncology (CSCO) have developed a set of lung cancer guidelines to promote standardization of lung cancer diagnosis and treatment in China. The CSCO guide references other guidelines and the latest updates in lung cancer research; it also has modified treatment guidelines for Chinese-specific populations; the goal is to benefit Chinese patients and offer practical instructions for doctors in China who treat patients with lung cancer.

This guide includes both tables and text but mostly tables with supplementary text descriptions. The information in the tables has been made as clear and concise as possible for convenient citation and reference, and evidence and consensus are included along with diagnosis and treatment suggestions. Addendums are attached to tables when needed. The CSCO categories of evidence and consensus are as follows: category 1 includes multicenter randomized controlled clinical trials and may vary between global and Chinese clinical practice; category 2 includes single-center randomized controlled clinical trials or highly influential translational medical research; category 3 includes studies that raised new questions. The text portions of this guide include detailed descriptions and reviews of the latest evidence, which are based on proof and academic findings to clarify current developments and to meet a higher level of clinical and academic needs. Novel drugs that are available in other countries but not yet approved in China are introduced in the text or summarized in separate tables.

The most significant characteristic of this set of guidelines is that it has two strategy levels focused on both resource availability and treatment value: a basic strategy and an optional strategy. Basic strategies are targeted to county-level hospitals and above. These recommendations are fundamental for diagnosis and treatment and are based on a high level of evidence and consensus; most importantly, they are accessible. Optional strategies are higher-level choices that include more effective treatments available in better medical centers in more developed regions. Our previous retrospective study of a large number of patients from an outpatient oncology database revealed major disparities in the treatment of patients with lung cancer in China. Therefore, it was important to develop new guidelines for treatment that are stratified according to available resources and treatment value.^11^ This stratification provides reasonable and actionable instructions for different levels of medical care providers, with the goal of tailoring the treatment of patients with lung cancer in China and reducing the burden of lung cancer.

Two examples can help explain the recommendation levels. First, for patients with stage IIIB NSCLC, the widely accepted standard treatment is definitive concurrent chemoradiation therapy.^12-17^ However, given the technical conditions necessary for radiotherapy and the capacity for treating radiation-related complications in China, the basic strategy in the CSCO guidelines only recommends a combination of radiotherapy and chemotherapy, either sequential or concurrent. For institutions fully qualified to provide radiotherapy, concurrent chemoradiotherapy is recommended as an optional strategy. A second example is bevacizumab combined with platinum-based chemotherapy as first-line therapy for metastatic NSCLC,^18-20^ which is a worldwide standard treatment strategy. However, the cost of the drug (¥20,000 to ¥35,000 per cycle for bevacizumab alone) is unaffordable for most families, so the CSCO guidelines list this as an optional strategy. Examples of first-line treatment of stage IV non–squamous-cell lung cancer without a driver gene are listed in Table 1.

**Table 1:**
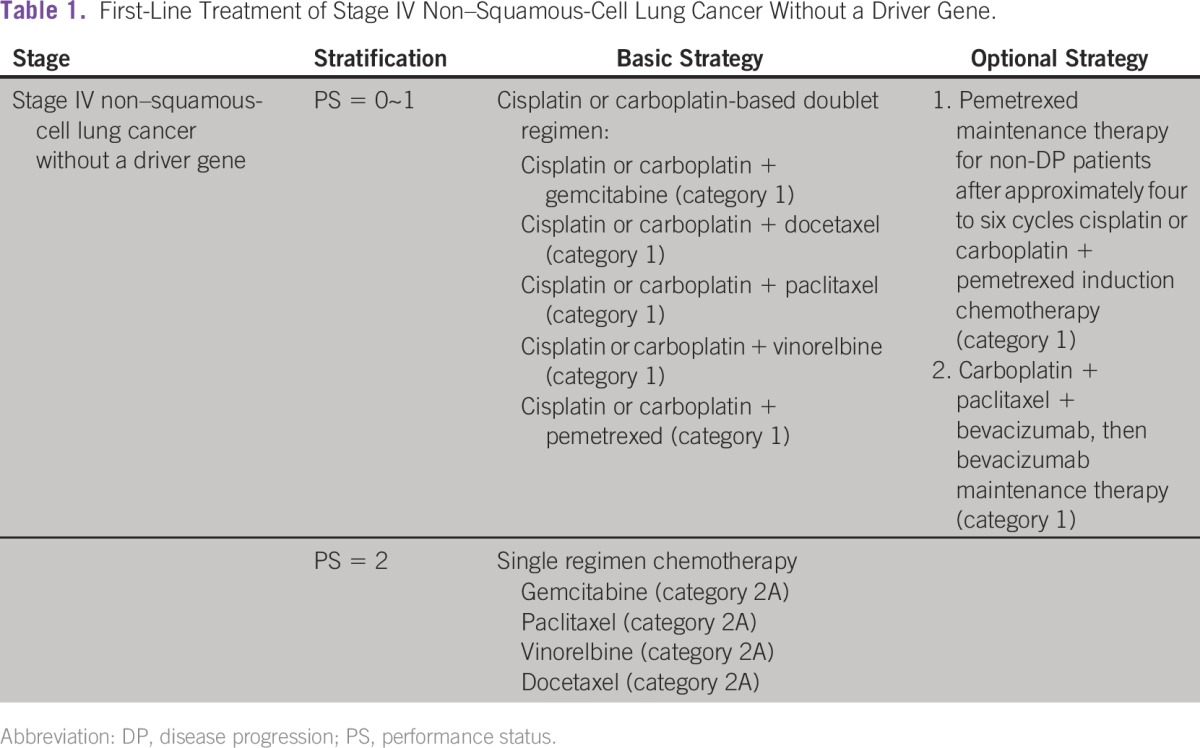
– First-Line Treatment of Stage IV Non–Squamous-Cell Lung Cancer Without a Driver Gene.

The latest discoveries and traits specific to Chinese people still need to be included in this guide, along with other topics such as palliative care. To keep up with the fast pace of research and discoveries in the field of lung cancer diagnosis and treatment, lung cancer experts from CSCO are scheduled to update the guidelines once a year, with a new guide planned for release each April. This is the first detailed set of lung cancer guidelines in China. With appropriate publicity and promotion, the guidelines are expected to improve the diagnosis and treatment of patients with lung cancer in China.

Increasing numbers of domestically produced novel drugs to treat lung cancer are being investigated and will eventually benefit Chinese patients with lung cancer. In addition, more and better individual-initiated trials are in progress, and academic organizations such as the Chinese Thoracic Oncology Group have achieved better outcomes among Chinese patients with lung cancer. Together, these activities will eventually improve diagnosis, treatment, and outcomes among patients with lung cancer in China.
